# Theta‐paced flickering between place‐cell maps in the hippocampus: A model based on short‐term synaptic plasticity

**DOI:** 10.1002/hipo.22743

**Published:** 2017-06-14

**Authors:** Shirley Mark, Sandro Romani, Karel Jezek, Misha Tsodyks

**Affiliations:** ^1^ Department of Neurobiology Weizmann Institute of Science Rehovot 76100 Israel; ^2^ HHMI Janelia Research Campus Ashburn Virginia 20147 USA; ^3^ Biomedical Center, Faculty of Medicine in Pilsen Charles University Pilsen 32300 Czech Republic; ^4^ Kavli Institute for Systems Neuroscience and Centre for Neural Computation, Norwegian University of Science and Technology Trondheim 7491 Norway

**Keywords:** attractor neural network, CA3, hippocampus, memory, place cell, recurrent neural network, teleportation, theta

## Abstract

Hippocampal place cells represent different environments with distinct neural activity patterns. Following an abrupt switch between two familiar configurations of visual cues defining two environments, the hippocampal neural activity pattern switches almost immediately to the corresponding representation. Surprisingly, during a transient period following the switch to the new environment, occasional fast transitions between the two activity patterns (flickering) were observed (Jezek, Henriksen, Treves, Moser, & Moser, 2011). Here we show that an attractor neural network model of place cells with connections endowed with short‐term synaptic plasticity can account for this phenomenon. A memory trace of the recent history of network activity is maintained in the state of the synapses, allowing the network to temporarily reactivate the representation of the previous environment in the absence of the corresponding sensory cues. The model predicts that the number of flickering events depends on the amplitude of the ongoing theta rhythm and the distance between the current position of the animal and its position at the time of cue switching. We test these predictions with new analysis of experimental data. These results suggest a potential role of short‐term synaptic plasticity in recruiting the activity of different cell assemblies and in shaping hippocampal activity of behaving animals.

## 1 INTRODUCTION

The hippocampus plays a critical role in spatial memory (Morris, Garrud, Rawlins, & O'Keefe, 1982; Nakazawa, McHugh, Wilson, & Tonegawa, 2004; Scoville and Milner, 1957). Neurons in the hippocampus fire at specific locations in the environment, the place fields (O'Keefe and Dostrovsky, 1971), and their activity is modulated by the ongoing theta rhythm (Buzsaki, 2002; Vanderwolf, 1969). The ensemble of active place cells in an environment defines a “map” of that environment (O'Keefe and Nadel, 1978). Partially overlapping populations of place cells are active in different environments (Muller and Kubie, 1987; Wills, Lever, Cacucci, Burgess, & O'Keefe, 2005). This phenomenon is referred to as global remapping (Fyhn, Hafting, Treves, Moser, & Moser, 2007; Leutgeb et al., 2005). The activation of a map is determined both by external sensory inputs (Muller and Kubie, 1987) and self‐motion cues from the medial entorhinal cortex (Fyhn et al., 2007; McNaughton et al., 1996; Wang, Romani, Lustig, Leonardo, & Pastalkova, 2015).

Jezek et al. (2011) examined the dynamics of global remapping in the CA3 region of the hippocampus following an abrupt switch of visual cues (“teleportation”). Two sets of visual cues elicited the activity of two different maps. During a few seconds following the abrupt switch in sensory cues, hippocampal activity transiently alternated between the two maps before settling into the new map (flickering). The transient nature of flickering suggests the presence of some form of short‐term memory. Following teleportation, the correlation between the instantaneous neural activity and the representation of the previously visited environment almost vanishes (Jezek et al., 2011), suggesting that short‐term memory cannot be maintained by reverberatory activity in CA3. These findings add to the evidence that place cell activity is not entirely driven by sensory cues but rather influenced by internally generated and history dependent activity (Cei, Girardeau, Drieu, Kanbi, & Zugaro, 2014; Diba and Buzsáki, 2007; Foster and Wilson, 2006, 2007; MacDonald, Lepage, Eden, & Eichenbaum, 2011; Pastalkova, Itskov, Amarasingham, & Buzsaki, 2008; Pfeiffer and Foster, 2013).

Internal representations of different environments and the spatial locations within the environment have been hypothesized to be stored in the form of attractor states in hippocampal circuits (McNaughton and Morris, 1987; Treves and Rolls, 1992; Tsodyks, 1999). According to the continuous attractor neural networks (CANN) modeling framework, each map is composed of labeled populations of neurons, where each neuron encodes a different position in the environment (Tsodyks and Sejnowski, 1995; Tsodyks, Skaggs, Sejnowski, & McNaughton, 1996). The synaptic strength between neurons decreases with the distance between the positions encoded by the neurons. This local excitation, together with long‐range inhibition, promotes the formation of a spatially localized activity profile on the map. CANN models can encode multiple spatial maps by superimposing synaptic structures related to place field locations in the corresponding environments. Global inhibitory feedback induces a competition between the maps (Battaglia and Treves, 1998; Monasson and Rosay, 2015; Samsonovich and McNaughton, 1997; Hedrick & Zhang, 2016).

In the CANN framework, the switch in sensory cues would cause the hippocampal model network to undergo a fast transition to the corresponding map, resulting in instantaneous remapping. The mechanism for reverse transitions (flickering) is less obvious. Flickering might be triggered by random fluctuations in population activity (Stella and Treves, 2011), but this would not account for the transient dynamics of flickering. To explain the transient nature of the flickering phenomenon, we considered CANN with short‐term synaptic plasticity (STP, e.g. Fung, Wong, Wang, & Wu, 2012).

There are several indications of STP presence in area CA3 of the hippocampus (Miles and Wong, 1986; Salin, Scanziani, Malenka, & Nicoll, 1996; Selig, Nicoll, & Malenka, 1999; Guzman et al., 2016). CANN with STP can account for several circuit dynamics observed in the hippocampus, such as phase precession, activity replays, and activity during the delay period of a spatial memory task (Romani and Tsodyks, 2015; Wang et al., 2015). In this contribution we show that CANN whose synapses are endowed with STP can account for the appearance of flickering events following the switch of environments. More specifically, the recurrent connections between neurons that were active in the previous environment remain temporarily facilitated following the switch in the cues. During a few theta cycles following the switch, the map of the new environment that receives stronger sensory inputs and the previously active map with facilitated recurrent connections compete via global inhibition. As a result, the previous map can be transiently reactivated due to theta modulations of population activity. We further test model predictions by analyzing data from Jezek et al. (2011).

## 2 METHODS

### 2.1 The model

**Figure 1:**
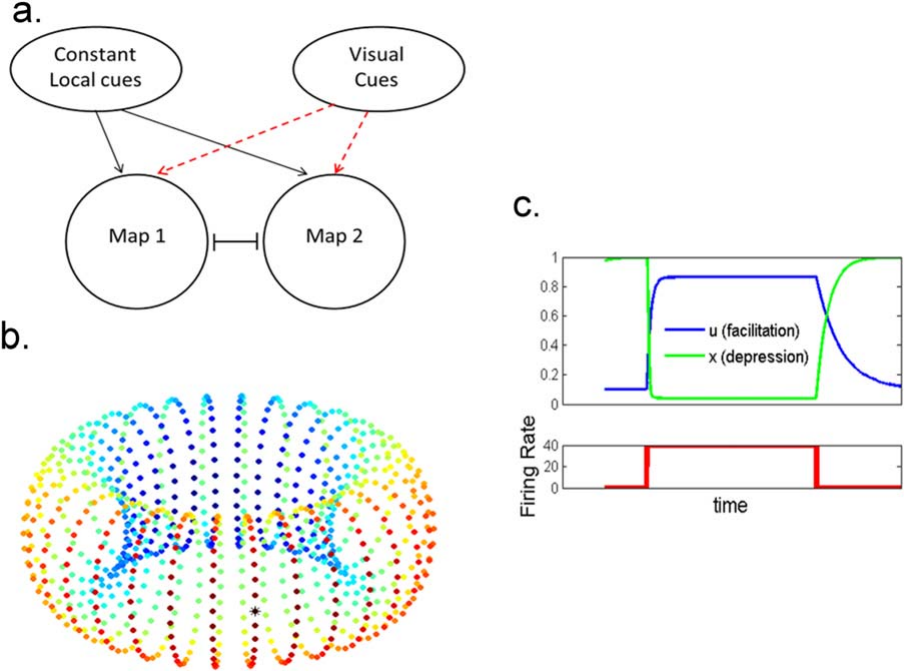
Schematic diagram of the model. (a) Two overlapping populations of firing rate units encoding maps of two environments. Upon switch of the external cues, part of the sensory inputs switches to the other map, while other localized cues are resistant to the switch. Both networks receive a spatially uniform theta‐modulated input. (b) Connectivity within a single population. Connectivity within a torus is dependent on the distance between the locations encoded by the units. Connectivity between tori is unstructured and inhibitory. The color code represents the synaptic strength between the unit denoted by the asterisk and all the other units that belong to the map (Blue, weak connections. Red, strong connections). (c) An example of the dynamics of a synapse endowed with short‐term plasticity. The amount of synaptic resources (*x*) decreases with increasing pre‐synaptic firing rate; the release probability (*u*) increases with firing rate. The product ( u⋅x) determines the overall modulation of the synaptic efficacy [Color figure can be viewed at wileyonlinelibrary.com]

We modeled the CA3 neural network as a network that stores the maps of two 2D environments (Figure 1a). To avoid complications due to boundary conditions, each environment was modeled as a torus of units with mutual inhibition between the tori. Each unit can be thought of as representing a pool of neurons with highly overlapping place fields. The similarity in the firing of place cells with nearby preferred locations allows for the definition of a firing rate (*m*), representing the average spiking activity of the pooled neurons. The connectivity between the units depends on the distance between the locations encoded by the units (Figure 1b) (Ben‐Yishai, Bar‐Or, & Sompolinsky, 1995; Conklin and Eliasmith, 2005; Pinto and Ermentrout, 2001; Romani and Tsodyks, 2010; Wilson and Cowan, 1973; Zhang 1996). Further, there are inhibitory connections between all units. Each unit receives a theta‐modulated input and a place specific input (see below). The network dynamics is described by the following equations

(1) $$\begin{array}{l} \tau \frac{dm_{i}^{}}{dt}=-m_{i}^{}+g\left(I_{{}_{i}}^{}(t)\right)\\ I_{{}_{i}}^{}(t)=\sum_{j=1}^{N}J_{\text{ij}}x_{j}(t)u_{j}(t)m_{j}(t)+I_{{}_{\text{ext}}}^{i}(t)+I_{0}\\ J_{\text{ij}}=\sum_{k=1}^{2}\xi_{i}^{k}\xi_{j}^{k}J_{1}(\cos(\varphi_{{}_{j}}^{k,1}-\varphi_{{}_{i}}^{k,1})+\cos(\varphi_{{}_{j}}^{k,2}-\varphi_{{}_{i}}^{k,2}))-J_{0}\\ g(z)=\alpha log(1+e^{\frac{z}{\alpha }}) \end{array}$$

where $m_{i}^{}(t)$ is the firing rate of unit *i* with two‐dimensional place field center $\left(\varphi_{{}_{i}}^{k,1},\varphi_{{}_{i}}^{k,2}\right)$ in map *k*. Each unit *i* in the network (*i = 1…N*) is characterized by a binary vector of selectivity for the two environments, $\xi_{i}^{k}$, where *i = 1…N* and *k =* 1,2. The selectivity for each environment (*k*) is assigned randomly from large pool of units such that $\xi_{i}^{k}=1$ with probability *f*, and zero otherwise (*f =* 0.25). In the Supplement Information Figure S12 we plot the dependence of the number of flickering events on *f*. Following assignment we only simulated the units that had assignment to one of the maps such each map contains exactly 2,500 units. With this choice of network size the linear spatial resolution in a map is $\frac{2\pi }{50}$ rad. τ is the integration time constant of the units (chosen to be of the same order of magnitude as the typical membrane time constant, tens of milliseconds), *J*_1_ is the synaptic efficacy of the distance dependent component in the network connectivity; *J*_0_ determines the strength of the uniform feedback inhibition (Figure 1b). We re‐analyzed the experimental data from Jezek et al. (2011) and checked whether the number of flickering events increases during each day. Increase in events number may imply learning and therefore not uniform and increased synaptic connectivity between the map. We did not find any increase in flickering events, therefore we conclude that there is no learning that connect the 2 maps (Supplamentary Information Figure S13). *I*_0_ is the background input. *g*(*z*) is the transfer function of the neurons; For large negative inputs, the firing rate increases exponentially with the input, while for large positive inputs *g*(*z*) is linear. α determines the width of the transition region between the exponential and the linear regimes. The shape of the transfer function may reflect heterogeneity in excitability of single neurons within a rate unit. The transfer function does not include a saturating non‐linearity at high input because the firing rate of the units are far from physiological saturation levels. The connections between the units are endowed with activity dependent short‐term synaptic plasticity (Figure 1c). Synaptic efficacy is modulated by the fraction of available synaptic resources (*x*) and the release probability (*u*). The release probability is increased (facilitated) every time a spike arrives; therefore it increases with presynaptic firing rate, while synaptic resources decrease following a spike and therefore decrease with presynaptic firing rate. In the absence of pre‐synaptic firing, *x* and *u* recover to their baseline values, *1* and *U*, with time constants τ_r_ and τ_f_, respectively (e.g. Tsodyks, Pawelzik, & Markram, 1998):

(2) $$\begin{array}{c} \frac{du_{i}}{dt}=\frac{U-u_{i}(t)}{\tau_{f}}+U(1-u_{i}(t))m_{i}(t)\\ \frac{dx_{i}}{dt}=\frac{1-x_{i}(t)}{\tau_{r}}-u_{i}(t)x{\left(t\right)}_{i}m_{i}(t) \end{array}$$

All units receive an external input, $I_{{}_{\text{ext}}}^{i}(t)$, which is composed of a theta modulated input, $I_{\theta }(t)=A_{\theta }\sin(2\pi f_{\theta }t)$, with amplitude *A*_θ_ and frequency $f_{\theta }$=*10Hz*, and a moving localized input.

(3) $$I_{\text{ext}}^{i}(t)=I_{\theta }(t)+(A_{\text{local}}^{i})\left([\cos{\left(\varphi_{{}_{i}}^{k,1}-\phi^{1}(t)\right]}_{+}+{\left[\cos(\varphi_{{}_{i}}^{k,2}-\phi^{2}(t))\right]}_{+}\right)+\eta_{i}^{}(t)$$

The amplitude of the localized input $A_{\text{local}}^{i}$ is composed of two terms; one denotes the contribution from external cues that do not change upon switching (*A*_1_) while the other originates from the cues (*A*_2_) that change upon teleportation, (Figure 1). Therefore the external cues are segregated into two types, depending on whether they are changed or maintained upon teleportation

$\left(\phi^{1}(t),\phi^{2}(t)\right)$ denotes the animal location, []_+_ is the linear threshold function. For units that belong to the map that encodes the current environment *A*_local_ *= A*_1_ *+A*_2_ while for the other units *A*local *= A*_2_.

In most network simulations we chose $\left(\phi^{1}(t)=\phi^{2}(t)\right)$ as the virtual animal trajectory. For the results presented in Supporting Information Figure S3, we used real animal trajectory that were taken from Jezek et al. 2011.

In some of the simulations (see results) we added colored noise (a time correlated noise) input $\eta_{i}(t)$ to the units (with time constant $\tau_{N}$ chosen to be the same as the time constant of integration of the rate units), that behaves according to the following equation:

(4) $$\tau_{N}\frac{d\eta_{i}}{dt}=-\eta_{i}+A_{n}\xi_{i}(t)$$

$\xi_{i}(t)$ is a spatially uncorrelated Gaussian white noise.

To identify flickering events in the noisy simulations, we band‐pass filtered the average network activity in the theta range (8–12 Hz) and segmented theta cycles based on the minima of the filtered network activity.

To examine the activity and synaptic efficacy of the units that encode the current location in each map, we averaged the firing rate and synaptic efficacy of units that have their place field center in the region defined by $\left(\cos(\varphi_{{}_{i}}^{1}-\phi^{1}(t))>0.5)\;and\;(\cos(\varphi_{{}_{i}}^{2}-\phi^{2}(t))>0.5\right)$.

Results in Figures 4, 5, 6 are obtained by averaging several realizations of the model.

All deterministic simulations have been calculated using MATLAB ode45 solver, that is, adaptive Runge‐Kutta. The simulations with noise have been calculated using Euler method with dt = 0.1ms. The parameters used in the simulations are written in table 1 and 2 (deviations from these parameters are mentioned in the legend of the relevant figure).

### 2.2 Analysis of the electrophysiological data from Jezek et al. (2011)

#### 2.2.1 Rate maps and flickering events definition

Data analysis was performed similarly to Jezek et al. (2011). Briefly, all the data was speed‐filtered such that only theta cycles in which the rat ran faster than 5 cm s^−1^ were included and tracking artifact were excluded (>100 cm s^−1^). Epochs longer than 0.05 s that did not include tracking data were excluded from the analysis. Rate maps with 30 × 30 spatial bins of 2 cm × 2 cm were created for each environment by calculating the firing rate of each recorded neuron within every bin, during the reference sessions, in which the animal walked in each environment without switching, and smoothing the map with a Gaussian filter (see end of Methods). During teleportation trials (trials that include the sudden switch of sensory cues) a vector of cell firing rates was calculated for each theta cycle (see Jezek et al., 2011 for details). A flickering event was defined as a theta cycle in which the activity vector during the cycle had significant low correlation with the rate map of the current environment and significant high correlation with the map of the other environment. The significance levels were determined by calculating a vector of cell firing rates for each theta cycle during the reference sessions and creating distributions of correlation coefficient values between these activity vectors and the rate maps of each environment. For each rate map, there are two different distributions; the first distribution corresponds to correlations with activity vectors of the same environment and the second distribution corresponds to correlations with activity vectors of the other environment. The threshold for low correlation was defined as the 5 percentile of the distribution corresponding to the current environment and the high threshold as the 95 percentile of the distribution corresponding to the other environment (see also Jezek et al., 2011).

We filtered the LFP in the theta band in order to estimate the activity vectors within individual theta cycles (as in Jezek et al., 2011). Briefly, the filter was constructed using a hamming window. Frequencies of 5 and 6 Hz were chosen for the low passband and stopband cutoff frequencies and frequencies of 10 and 11 Hz for the high passband and stopband cutoff frequencies. Theta phase of minimum activity was found by assigning a phase from 0° to 360° to each spike. The phase assigned to each spike was interpolated linearly according to the times of successive peak and trough and the spike time (every interpolation was in the range [0,180] degrees). The phase with the minimal firing rate was chosen for segregating the signal into theta cycles.

#### 2.2.2 Correlation coefficient between the number of flickering events and theta power or average distance

To assess theta power (5–10 Hz) during periods of increased flickering probability (see Results), average theta power was calculated for each period between 250 ms before the first network transition to the correct representation and 5 s after the transition (the results shown in Figure 5 are robust to changes in the definition of this period, see Supporting Information Figure S4). Theta power of the un‐filtered EEG signal was normalized by the wide band power (1–125 Hz, results were unaffected by the chosen normalization band). According to our model, the probability of observing a flickering event depends on the distance between animal position at network transition time (referred to as “switching position”) and its current position. Therefore, we examined the correlation between the number of flickering events and this average distance (see Results). Average distance from the switching position was calculated over the 5 s that follow network transition. As mentioned above we only considered epochs with rat speed higher than 5 cm s^−1^. During the recordings there are short epochs with tracking artifact and low animal velocity. Hence, during the epochs around the switch there are short time bins in which flickering cannot be estimated. To overcome the resulting bias for low number of flickering events (during trials with larger number of such bins) we normalized the number of flickers to the relevant time interval:

$$n_{\left[\frac{\text{flicker}}{s}\right]}=\frac{N_{\text{flickers}}}{(1-p_{\text{lowV}})T}$$

*N*_flickers_ is the number of flickering events; *p*_lowV_ is the fraction of time bins with tracking artifact or of low velocity during the tested epoch (*T*). We ignore switch trials with high percentage (>50%) of theta cycles with low velocity or tracking artifact.

Theta power and the average distance from switch positions are correlated, therefore, in order to estimate the correlation between the number of flickering events and those variables we calculated the partial correlation coefficient (Howell, 2009). The flicker number, theta power and average distance are not normally distributed; hence, the *p* values for the partial correlation coefficient were calculated by constructing shuffled distributions of correlation coefficients. For each shuffle, we permuted the vector of flickering events number, while keeping the pairs of distance and theta power, such that the correlation between these two variables remains. While calculating the partial correlations we included the average firing rate in the interval around the stimulus switch (defined above) as a control variable.

Gaussian filters’ weights:

GF = [0.0025 0.0125 0.0200 0.0125 0.0025;…
0.0125 0.0625 0.1000 0.0625 0.0125;…
0.0200 0.1000 0.1600 0.1000 0.0200;…
0.0125 0.0625 0.1000 0.0625 0.0125;…
0.0025 0.0125 0.0200 0.0125 0.0025]

## 3 RESULTS

Following previous studies (Battaglia and Treves, 1998; Romani and Tsodyks, 2015; Samsonovich and McNaughton, 1997; Tsodyks, 1999; Tsodyks et al., 1996), we modeled a hippocampal place map as a manifold of units, with each unit representing the average firing rate of neurons with highly overlapping place fields. The recurrent connections between the units decay with the distance between the locations encoded by the units (Figure 1b). The connections between the units are symmetric such that a continuous attractor is formed (Ben‐Yishai et al., 1995; Tsodyks, 1999; Tsodyks et al., 1996). Each position is encoded by a “bump” of activity on the manifold. Two maps of different environments are modeled as two overlapping populations of units that compete through global inhibition (Tsodyks, 1999). The synaptic connections are endowed with activity dependent short‐term plasticity (STP, Figure 1c). The units receive theta‐modulated input and spatially localized inputs. The localized input to the units is of two types: visual cues that are changed immediately as the environment is changed, and other external cues such as olfactory cues and motion integration (Figure 1a) that are stable upon switching.

### 3.1 Flickering occurs after switching

**Figure 2:**
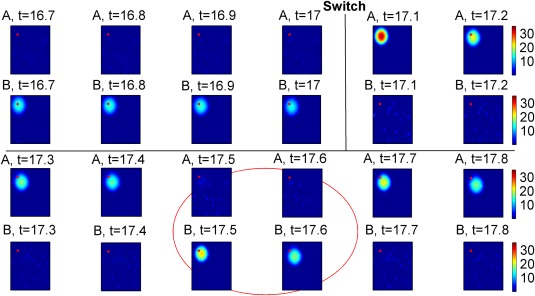
Flickering in networks with short‐term synaptic plasticity. Network activity dynamics. Each panel shows the activity of each unit in a map (single pixel), averaged across a theta cycle (colorcode: firing rate (Hz), first and third rows: map A, second and fourth rows: map B). Flickering events can be observed following the switch of external cues (red circle, *t* = 17.5s, 17.6 s). The red dot denotes the location of the virtual animal (peak external input). Note that the simulations do not include a noisy input [Color figure can be viewed at wileyonlinelibrary.com]

In the model, similar to the experiment, switching the visual input results in an almost immediate transition of activity from one map to the other. During most theta cycles the activity of the population that encodes the new environment is larger than the activity of the other population. During a brief period following network transition to the current representation we observed several theta cycles in which the activity of the population that represents the previous environment becomes larger (flickering event, Figure 2, Figure 4a; for other distance dependent connectivity matrix see Supporting Information Figure S1, for a real animal trajectory see Supporting Information Figure S3, 4 and S5).

### 3.2 Flickering results from short‐term synaptic plasticity

During each cycle of the external oscillatory theta input there is a competition between the two populations due to global inhibition and the connectivity within each map. The input to the populations and the network's gain, which depends on the strength of the synaptic connections, determine which map wins the competition. Without short‐term synaptic plasticity both populations have the same gain, therefore the input strength alone determines which population would win and flickering is not expected (unless strong enough noise is present, see below). In the presence of STP, the recurrent connections between neurons encoding the previous environment remain temporarily facilitated following the switch in the cues (Figure 4a, Supporting Information Figure S2). As a result, the previous map can win the competition and be transiently reactivated.

**Figure 3:**
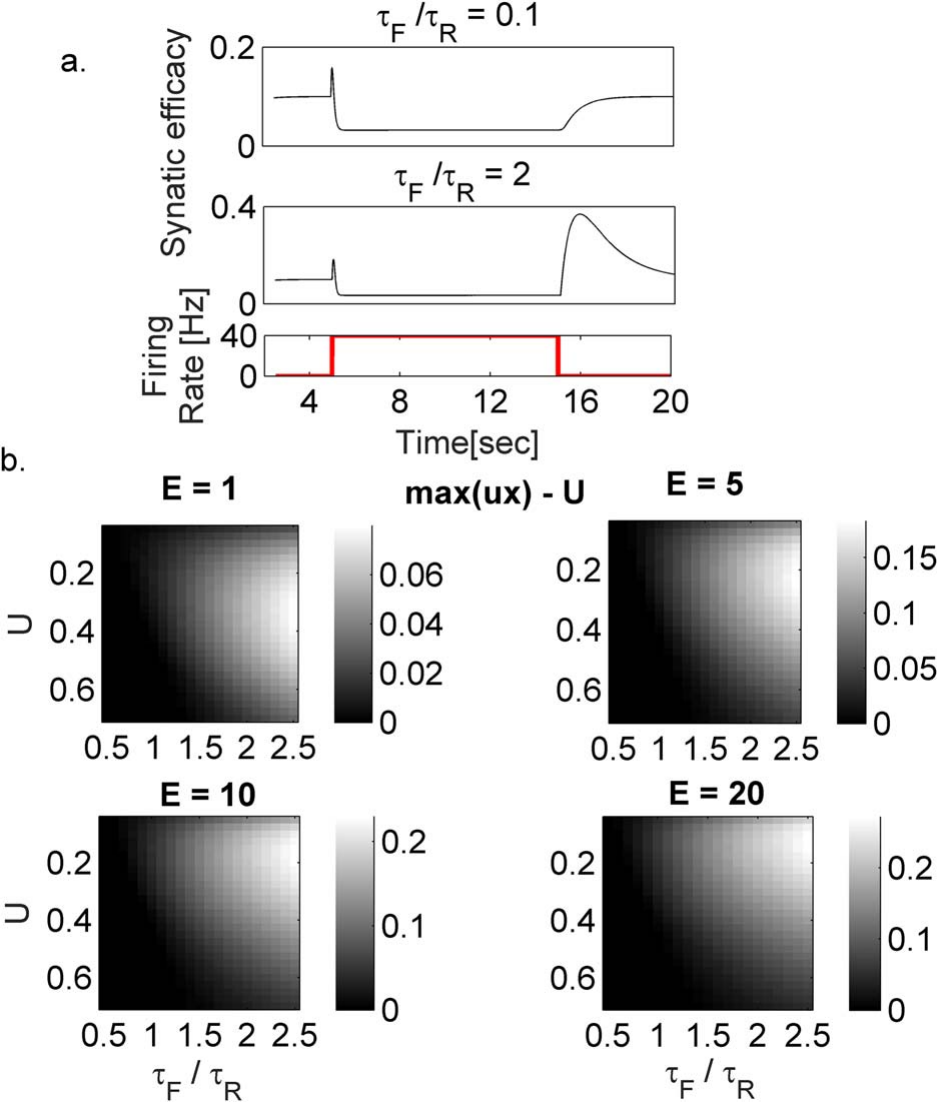
Synaptic rebound dynamics. (a) Synaptic efficacy (ux) changes following a transient input. Note the transient increase of synaptic efficacy following a sharp increase or decrease in presynaptic firing rate (synaptic rebound). The magnitude of the rebound increases with the ratio between facilitation and depression time constants (top vs. middle panels). (b) Rebound (maximal efficacy (ux) ‐U) response to a firing rate pulse (2 s). Each panel shows the rebound amplitude (color coded) for different facilitation/depression time constant ratios and baseline release probability U. Different panels: different firing rate pulse amplitudes (E). Higher ratio between facilitation and depression time constants results in larger rebound response. Note that the dependency on the baseline release probability U is nonmonotonic. An increase in the firing rate pulse produces a larger activation of the synapses resulting in a higher rebound [Color figure can be viewed at wileyonlinelibrary.com]

To characterize the contribution of STP to flickering, we first examined the dynamics of a single synapse in response to a pulse in pre‐synaptic firing rate. A sharp increase in firing rate produces a transient increase of the synaptic efficacy (*ux*). Following stimulus termination, the synaptic resources (*x*) recover faster than the relaxation of the release probability (*u*). Hence, a synaptic rebound appears, the synaptic efficacy grows before decaying to the steady state value (Figure 3a, middle panel). The synaptic rebound arises from short‐term synaptic facilitation, as decreasing the ratio between facilitation and depression reduces rebound amplitude (Figure 3b). The dependency on the baseline release probability (*U*) is non‐monotonic; the difference between the maximal synaptic efficacy and the baseline reaches maximal values for intermediate baseline utilization values (Figure 3b). The synaptic rebound depends on the presynaptic firing rate: A higher firing rate results in larger synaptic modification that will be followed by a higher rebound (Figure 3b).

**Figure 4:**
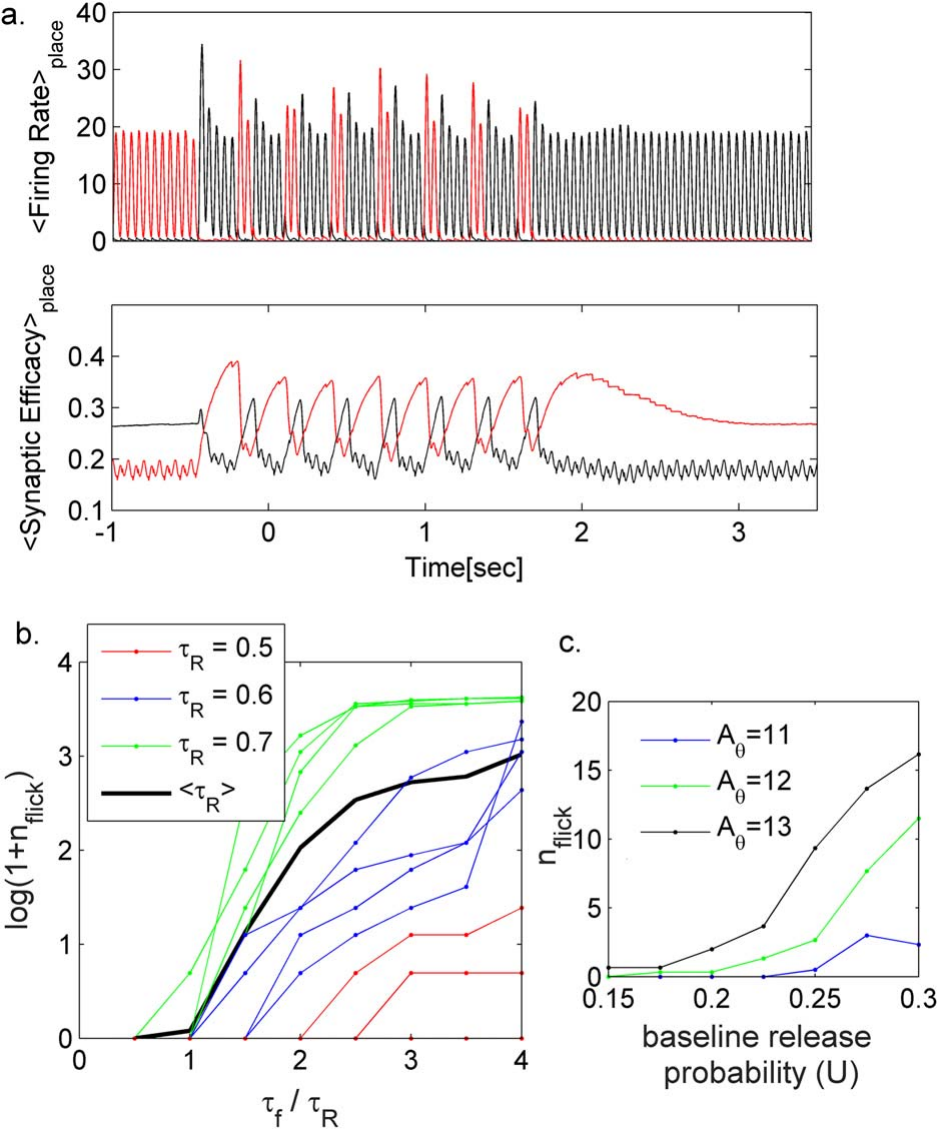
**Effect of short‐term synaptic plasticity on the occurrence of flickering events.** (a) Average firing rate of the units that encode the current location (upper panel, see methods) and corresponding average synaptic efficacies (lower panel), red—units in the previously active population, black—units in the population encoding the new environment. The occurrence of flickering events depends on the difference between the synaptic efficacies (lower panel) in the two populations. *A*_θ_ = 12 Hz other parameters as in Figure 2. (b) Influence of the ratio between facilitation and depression time constants on the number of flickering events. Black line, average number of flickering events over different $\tau_{r}$( $0.6\leq \tau_{r}\leq 0.8$). Colored dots: single simulation results; each color corresponds to different $\tau_{r}$. A_θ_ = 12Hz, other parameters as in Figure 2. (c) Effect of baseline release probability U on the number of flickering events for different amplitudes of theta modulation (see also Supporting Information Figure S11) [Color figure can be viewed at wileyonlinelibrary.com]

In a network, a stronger and longer‐lasting synaptic rebound following stimulus offset, compared to the initial response to stimulus onset, would increase the gain of the previously active population compared to the gain of the new one (Supporting Information Figure S2), therefore we expect a tendency of the network to produce a flickering event. To confirm this we examined the average activity and the synaptic efficacy of the units that encode the current location in each map (Figure 4a, see Methods); upon network transitioning to the map that represents the current environment, the difference between the rebound response of the synapses in the previously active map and the initial response of synapses in the currently active map increases until a flicker event is generated and subsequent events can take place until the network reaches a steady state (Figure 4a). Note that each flickering event could produce an additional synaptic rebound and thus allows the network to produce additional flickering events. This mechanism allows the network to transiently sustain flickering for time scales exceeding any time scale of the system. It should be noted that there is a parameter regime in which baseline flickering exists (Supporting Information Figure S7).

The occurrence of flickering events is a robust phenomenon of the model that does not require fine‐tuning of the parameters (Figure 4). The magnitude of the rebound response depends on synaptic parameters (Figure 3b), in particular, on the ratio between facilitation and depression time constants (*T*_f_/*T*_r_, Figure 3a), and the level of synaptic activation (i.e., firing rate, Figure 3b). Hence, the number of flickering events depends both on synaptic parameters (Figure 4b,c, Supporting Information Figure S11) and network activity.

A closer look at the dynamics during flickering reveals that at the beginning of each theta cycle (Figure 4a) the activities of both populations grow until the activity of one population dominates over the other. Hence, states in which both populations are simultaneously active (mixed states) can appear at the beginning of theta cycle but not at the end, as observed in the experimental data (Hasselmo, Clara, & Bradley, 2002; Jezek et al., 2011; Redish, 1999; Redish and Touretzky, 1998). Note that the competition between the attractors is governed by the difference in the localized external inputs to the maps. Decrease in this difference results in increase number of flickering events (Supporting Information Figure S8).

### 3.3 Flickering probability is affected by theta power and animal position

**Figure 5:**
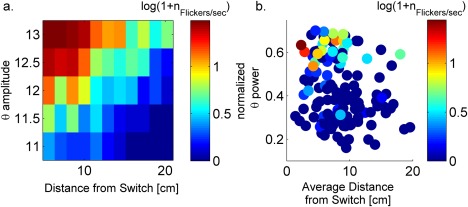
Flickering depends on theta power and animal position after the switch in environment. (a) The number of flickering events ( $n_{\text{Flicker}}$) increases with theta amplitude and decreases with distance from switch position ‐ model. Each square shows the number of flickering events during 5s after the switch (color coded), from a simulation with different theta amplitude ( $\frac{A_{\theta }}{\left|I_{0}\right|}$) and virtual animal speed (constant during the simulation), which results in a different average distance from the switch position during 5 s following the switch. (b) Data: Each dot represents the number of flickering event per seconds in a time window of 5s after the switch of sensory cues (color coded,) from a single trial, characterized by (i) The average distance of the animal position in the time window (ii) Normalized theta power around the switch. Partial correlation coefficient between theta power and $n_{Flic\ker}$ was 0.44 with *p* value < 0.001. Partial correlation coefficient between V and *n* Flickers was −0.15 with *p* value<0.05 (see methods) [Color figure can be viewed at wileyonlinelibrary.com]

According to our model, the increase in flickering probability for a few seconds following the switch in the environment results from STP. In the map corresponding to the old environment, synapses between the units encoding the recently active place fields are facilitated. Hence, we expect an increased flickering probability the closer the virtual animal is to the place field of the units that were recently active in the old environment, such that there is an overlap between the place cells that receive localized external input and the place cells with increased synaptic efficacies (model, Figure 5a). The animal should be close to its previously visited position during short time interval following the switch; otherwise the synaptic memory will decay.

In simulations with high amplitude oscillatory theta input, as in the results described above (Figure 2), the activity of all units decreases once every cycle. This decrease in activity reduces global inhibition and therefore resets the competition from one cycle to the next, allowing the reactivation of the old environment. With lower theta amplitude, network activity may not decrease enough to reset the competition. Further, as mentioned above, the higher the activity the higher the tendency to produce a flickering event. The peak activity in the network increases with theta power. Hence, stronger theta would result in higher tendency to produce flickering events following a switch of the environment.

In summary, our model predicts that the number of flickering events should (i) decrease with the distance that the animal travels after the switch in environments and (ii) increase with the theta power during the time window near the switch (Figure 5a). We analyzed the experimental data (Jezek et al., 2011) and estimated the number of flickering events as a function of the average distance from the switching position (a small distance would imply that the animal stayed close to the previously active place fields in the old environment). We also estimated the normalized theta power during epoch of several seconds around the switch (see Methods). We observed that the number of flickering events increases with theta power and decreases with the average distance (data, Figure 5b, Supporting Information Figures S6 and S9), in agreement with the prediction of the model (partial correlation coefficient between the theta power and the number of flickering events is 0.44, *p* < 0.001 ( $p=2\cdot 10^{-16}$) and partial correlation coefficient between the average distance and the number of flickering events is *−*0.157, *p* < 0.05 ( p=0.027). It is important to note that average velocity and distance are strongly correlated, *c* = 0.64, $p=10^{-17}$, therefore it is not possible to disambiguate their contribution to flickering (Supporting Information Figure S9, Figure S10).

### 3.4 The role of noise in flickering

**Figure 6:**
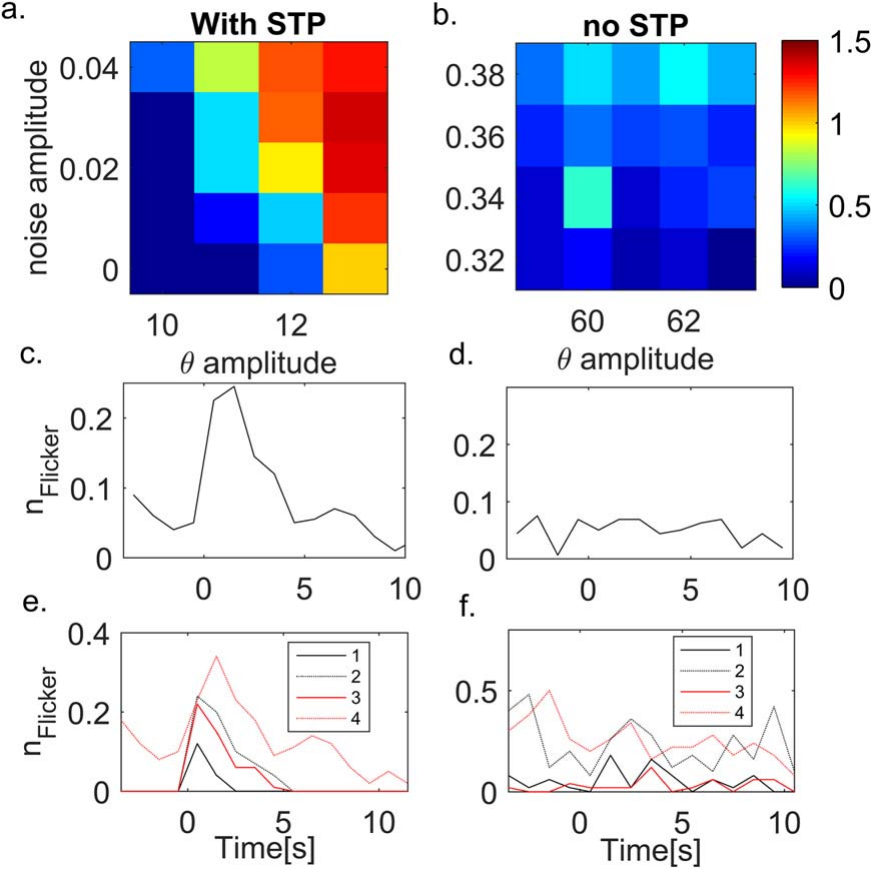
Noise increases flickering probability. (a) Average number of flickering events (over five different realizations) for different amplitudes of theta modulation and noise strength. Network with STP in the synaptic connections (parameters as in Figure 2, see Table 1). (b) As in (a), but the synapses in the network have no STP. (c) Time course of the number of flickering events (averaged over two different noise values, *A*_n_ = 0.01, 0.025, and 5 network realizations) for a network with STP in the synaptic connections. The probability of flickering exhibits a transient increase as a result of STP. *A*_θ_ = 12 Hz, all other parameters are the same as in Figure 2. (d) Time course of the number of flickering events in a network without STP in the synaptic connections. The probability of flickering events is approximately constant throughout the simulation. Parameters: *A*_n_ = 0.3, 0.4. (e) Network with STP: time course of the number of flickering events for different noise and theta amplitudes (averaged over five realizations). Higher theta and noise amplitude results in higher probability for flickering events and longer transient increase in flickering probability. (3) *A*_θ_ = 12 Hz, *A*_n_ = 0.01 (4) *A*_θ_ = 12 Hz, *A*_n_ = 0.025 (1) *A*_θ_ = 11 Hz, *A*_n_ = 0.01 (2) A_θ_ = 11 Hz, *A*_n_ = 0.025. (f) As in (e) for a network without STP. Flickering probability is constant in time for all noise and theta amplitudes. *A*_θ_ = 62 Hz, *A*_n_ = 0.3, *A*_θ_ = 62 Hz, *A*_n_ = 0.4, *A*_θ_ = 60 Hz, *A*_n_ = 0.3, *A*_θ_ = 60 Hz, *A*_n_ = 0.4 [Color figure can be viewed at wileyonlinelibrary.com]

As discussed above, a lower theta amplitude results in a reduction of the number of flickering events and even their disappearance (Figures 5 and 6a). The presence of noise in networks that would otherwise exhibit no flickering events may increase the range of parameters in which flickering can occur (Figure 6a). The noise may originate from several sources (Faisal, Selen, & Wolpert, 2008).

Noise may also induce flickering in a network without STP. In this network, as in a network with synaptic connections that are endowed with STP, the overall number of flickering events increases with the amplitude of the fluctuating input and theta amplitude (Figure 6b). Note, however that flickering probability is approximately constant and exhibits no dependence on teleportation (Figure 6d), in contradiction to experimental observations. The presence of STP results in a transient increase of the flickering probability following teleportation (Figure 6c). The transient period of increased flickering probability depends both on theta and the noise amplitude (Figure 6e), the noise enables the occurrence of flickering events when the gain of the previous map is not large enough to enable flickering by itself. Further, noise and theta increase the baseline flickering probability in both models (Figure 6e,f).

## 4 DISCUSSION

We developed a model that suggests a mechanism for the flickering in CA3 activity patterns between two maps following a fast switch of sensory cues. We further test predictions of the model. Jezek et al. (2011) showed that during most theta cycles there is a period in which only one map is active; therefore, the existence of flickering events suggests the presence of short‐term memory not mediated by neuronal activity in CA3. In our model short‐term memory is being held by short‐term plastic synapses (see also Mongillo, Barak, & Tsodyks, 2008). We chose to model hidden short‐term memory using a synaptic mechanism, though we cannot exclude other forms of short‐term memory such as intrinsic adaptation/facilitation.

The model is a continuous attractor neural network composed of two overlapping populations that compete with each other as a result of global inhibition. A stimulus to one of the populations, the one representing the map of the current environment, results in the activation of that map. Following a switch in the external inputs to the other map, the activity can fluctuate back and forth between the maps (flickering) until it converges to the map that represents the current environment. The flickering occurs as a result of a temporarily increased gain of the previously active map due to short‐term synaptic plasticity, which induces the competition between the maps.

Each flickering event results in the activation of the previously active map and therefore affects the short‐term dynamics of synapses in that map. Following the subsequent reactivation of the map that represents the current environment, the effective synaptic efficacy of the other map increases again as a result of STP rebound. This alternating process enables the occurrence of several flickering events, during a time period which can be longer compared to the time constants of the neurons and synapses. We further examined the effect of theta power and animal distance from its position at the time of the switch both in the experimental data and in the model. We showed that a smaller average distance from the switch position and higher theta power results in an increased number of flickering events.

We examined the dependence of flickering on synaptic release probability and synaptic time constant. We predict that manipulating calcium dynamics in the synapses will affect flickering dynamics. Further, in our model, as a feature of competitive attractor neural network, the similarity of the external inputs between the two environments will affect the number of flickering. We therefore predict that influencing the similarity of the two environments for example, by choosing environments from a morphed sequence (Wills et al., 2005) will shape the number of flickering events.

A possible alternative explanation for the flickering phenomenon is that flickering events are inherited from external inputs as a result of sensory cues that are common to both environments together with noise (Figure 6b). However, fluctuation in sensory input would not account for the transient increase in flickering probability after teleportation (Figure 6c,d).

Flickering was also observed in a neural network with one population of neurons that encodes multiple environments (Monasson and Rosay, 2015). The purpose of that study was to examine the mechanism of spontaneous transitions (flickering) between the two representations, but the temporal dynamics of the flickering probability was not discussed.

Place cells integrate external sensory inputs with path integration cues from entorhinal cortex (McNaughton et al., 1996; Touretzky and Redish, 1996). During the switch between environments the animal remains at the same arena, hence, the path integration is not disrupted by the switch. An alternative model may involve the dynamics of the path‐integration inputs. A scenario in which the grid network does not remap following the switch but remaps after integration of hippocampal inputs, could result in a transient increase of flickering probability following the switch due to decreased difference between the external inputs to the two maps as long as there is no remapping in EC. This model would not explain the dependency of flickering on the animal location in the arena and it is unclear how it could account for the time‐scale of increased flickering (several seconds) following the switch (an analysis of this modeling scenario is outside the scope of this work).

Previous theoretical studies reinforce the role of short‐term synaptic plasticity in the hippocampus of behaving animals. Continuous attractor models with dynamical synapses can account for several observations of place cell dynamics such as phase precession, sharp waves and activity replays (Romani and Tsodyks, 2015), and the dynamics of episode (or time) cells in the hippocampus (Gill, Mizumori, & Smith, 2011; MacDonald, Carrow, Place, & Eichenbaum, 2013; Pastalkova et al., 2008; Wang et al., 2015). Short‐term synaptic plasticity may also assume a role in stabilizing circuit dynamics. Inhomogeneities in the synaptic connections or synaptic depression mechanisms may result in a fast drifting of the localized activity bump (Tsodyks et al., 1996; York and Van Rossum, 2009). It is natural to assume that the connectivity in the hippocampus is not perfectly tuned; therefore, a mechanism for slowing down the drift of the activity bump is important. Synaptic facilitation may be a good candidate mechanism for slowing down the drift (Itskov, Hansel, & Tsodyks, 2011). The agreement of our model with the experimental results and the previous modeling efforts point to short‐term synaptic plasticity mechanism as a strong determinant in the recruitment of different cell assemblies in hippocampal circuits.

The functional relevance of flickering to hippocampus encoding is an open question. Hippocampus is involved in the process of choosing the right context to reach a decision (Dupret, O'neill, & Csicsvari, 2013; Jackson and Reddish, 2007; Kelemen and Fenton, 2010;) and planning an action, which may be mediated by “mental time travel” (Botzung, Denkova, & Manning, 2008; Hopfield, 2010; Pfeiffer and Foster, 2013; Suddendorf and Corballis, 2007; Wikenheiser and Redish, 2015). It is now being established that the hippocampus represents different environments or context with orthogonal cells assemblies (Fyhn et al., 2007; Malvache, Reichinnek, Villette, Haimerl, & Cossart, 2016; Wills et al., 2005). When the external cues for the correct context are ambiguous, hippocampus may alternate between possible representations (cell assemblies) in order to facilitate the selection of the right context for the task (see also Savin, Dayan, & Lengyel, 2014). It is possible that the occurrence of flickering reflects a state that favors mental exploration. In this state, network parameters are adjusted such that the probability to switch between the different representations increases. The increased flickering probability enables “reexamination” of the alternative contexts by downstream areas (Botzung et al., 2008) and reflects higher flexibility of CA3 network during the process of context selection. The activity in those areas may shift hippocampal activity to the other, alternative context. Further, our model suggests that controlling network parameters such as the synaptic properties (by different neuromodulators), or modulating theta input may shift the tendency of the hippocampus to wander between different representations.

**Table 1:** Parameters for the model with STP (Figure 2)

τ (s)	0.01
*U*	0.25
τ_r_ (s)	0.6
τ_f_ (s)	1.9
*A*_1_ (Hz)	4
*A*_2_ (Hz)	0.5
*A*_θ_ (Hz)	13
*V* (rad s^−1^)	2π/10
*J*_1_	$\frac{14\cdot 2\pi }{N}$
*J*_0_	$\frac{-18\cdot 2\pi }{N_{}}$
*I*_0_ (Hz)	−1
α	1

**Table 2:** Parameters for the model without STP

τ (s)	0.01
*A*_1_ (Hz)	3.25
*A*_2_ (Hz)	0.75
*A*_θ_ (Hz)	60
*V* (rad s^−1^)	2π/10
*J*_1_	$\frac{35\cdot 2\pi }{N}$
*J*_0_	$\frac{-42\cdot 2\pi }{N_{}}$
*I*_0_ (Hz)	−1
α	1

## Supporting information

Supporting Appendix 1

Supporting Appendix 2

Supporting Figure 1

Supporting Figure 2

Supporting Figure 3

Supporting Figure 4

Supporting Figure 5

Supporting Figure 6

Supporting Figure 7

Supporting Figure 8

Supporting Figure 9

Supporting Figure 10

Supporting Figure 11

Supporting Figure 12

Supporting Figure 13
