# Supplementary material for: Theta‐paced flickering between place‐cell maps in the hippocampus: A model based on short‐term synaptic plasticity
Source: Hippocampus. 2017 Jun 14;27(9):959–70. doi: 10.1002/hipo.22743 (PMC5575492; doi:10.1002/hipo.22743)
Supplement: Supplementary file 2 — Supporting Appendix 2 [file HIPO-27-959-s002.pdf]

Supplementary material

Connectivity profile

In the main text we reported simulation results from networks whose synaptic weights were chosen according to a cosine-shaped function of the distance between the units. Here we show that the results are robust to changes in the choice of connectivity profile. We examined the dynamics of a network with synaptic strengths decaying with distance between the units according to a generalized Von-Mises (e.g. Wang et al. 2015), see Fig. S1.

Fig S1. **Flickering in a network with short-term synaptic plasticity does not depend on the specific connectivity matrix.** Network activity before and after a switch in sensory cues in a 2D network with mixed selectivity units (format as in Fig. 2). The connectivity strength decays with the distance between the units according to the generalized Von-Mises function: $W_{ij} = e^{k(\cos(\theta_i^1 - \theta_j^1) + \cos(\theta_i^2 - \theta_j^2))}$. Parameters: k = 0.3, $J_0 = \frac{-20 \cdot 2\pi}{N}$, $A_\theta = 18Hz$, all other parameters as in Figure 2 (no noisy input present).

Fig. S2. **Synaptic efficacy during network transitions**. Synaptic efficacy time course in the entire network. Format as in Figure 2, but here pixel color denotes the synaptic efficacy (ux) from corresponding pre-synaptic neurons. Note the increased synaptic efficacy of the units in the non-active map during the theta cycle before the first flickering event (t = 17.5sec).

Fig S3. **Example of real animal trajectory** during day 1 of the experiment.

Fig S4. **Average activity following switch in sensory cues** – real animal trajectory.

In order to make sure that our results are not affected by the virtual animal trajectories that we chose, we simulated a virtual animal that moves according to real animal trajectories (taken from Jezek et al. 2011).

a) Activity of the units around the location of the peak external input (as in Fig. 4a).
b) Real animal speed.
c) Synaptic efficacy following the switch.

d) Real animal trajectory, normalized to be between [0,2π]. Colorcode -time. Red dot – switching position.

Fig S5. **Number of flickering event as a function of distance – real animal trajectory.**

We simulated a virtual animal running real rats trajectories which were taken from Jezek. et al 2011. (see figure S3 for example). We simulated trajectories of -10sec to +10sec around all switches and calculated the number of flickering events and averaged animal distances following the switch. Using analysis of covariance, we quantified the slops of the number of flickering to distance dependencies and their significant (for both theta values the p-values<<0), the intercept of the two line are also significantly different (F=40.2, p-value<<0).

Fig. S6. **Flickering probability as a function of theta power and distance by using different time windows**.

a. Time before switch = 0.25sec, time after switch 7sec, $P_{value-theta} < 0.001, P_{value-distance} = 0.067$
b. Time before switch = 0, time after switch 8sec, $P_{value-theta} < 0.001, P_{value-distance} = 0.04$
c. Time before switch = 0, time after switch 4sec, $P_{value-theta} < 0.001, P_{value-distance} = 0.054$
d. Time before switch = 0, time after switch 5sec, $P_{value-theta} < 0.001, P_{value-distance} = 0.03$

Fig S7. **Sustained Flickering events**.

a. A regime in which baseline number of flickering exists also in the deterministic model. The color-code represent the regime, black – no flickering, gray – flickering just during the first lap, white – flickering continues to the second round and sustain for the entire 20sec of simulations.
b. The ratio between the number of flickering events at the second and first round (last 10sec to first 10sec); Note transient increase in flickering probability following the teleportation.

Fig S8. **Number of flickering events as a function of the difference between external inputs to the two maps.** Once the difference in external stimulus is large enough such that a switch in

the representation occurs, the number of flickering events increases as the difference between external inputs decreases.

Fig S9. **Number of flickering events plotted separately as a function of theta power and distance**. (Time before switch = 0.25sec, time after switch 5sec).

Fig S10. Number of flickering events as a function of velocity. $P_{value} = 0.03$.

FigS11. Non-monotonic relation between the number of flickering events and the utilization parameter.

FigS12. **The number of flickering events as a function of the overlap between the maps.** (parameter *f*, all the other parameters as in main text figure 2).

FigS13. **Number of flickering event as a function of switch time (**re-analyzing experimental results from Jezek et al. 2011)**.**

The number of flickering events does not depend on switch time during the day. Therefore, there is no learning that may lead higher probability to flicker.

a. Number of flickering as a function of switch time in each day. Black bold line – average over days.
b. Cumulative function of the number of flickering as a function of switch time, a linear trend can be observed. Black bold line – average over days.
