# Supplementary figures and images for: Theta‐paced flickering between place‐cell maps in the hippocampus: A model based on short‐term synaptic plasticity
Source: Hippocampus. 2017 Jun 14;27(9):959–70. doi: 10.1002/hipo.22743 (PMC5575492; doi:10.1002/hipo.22743)

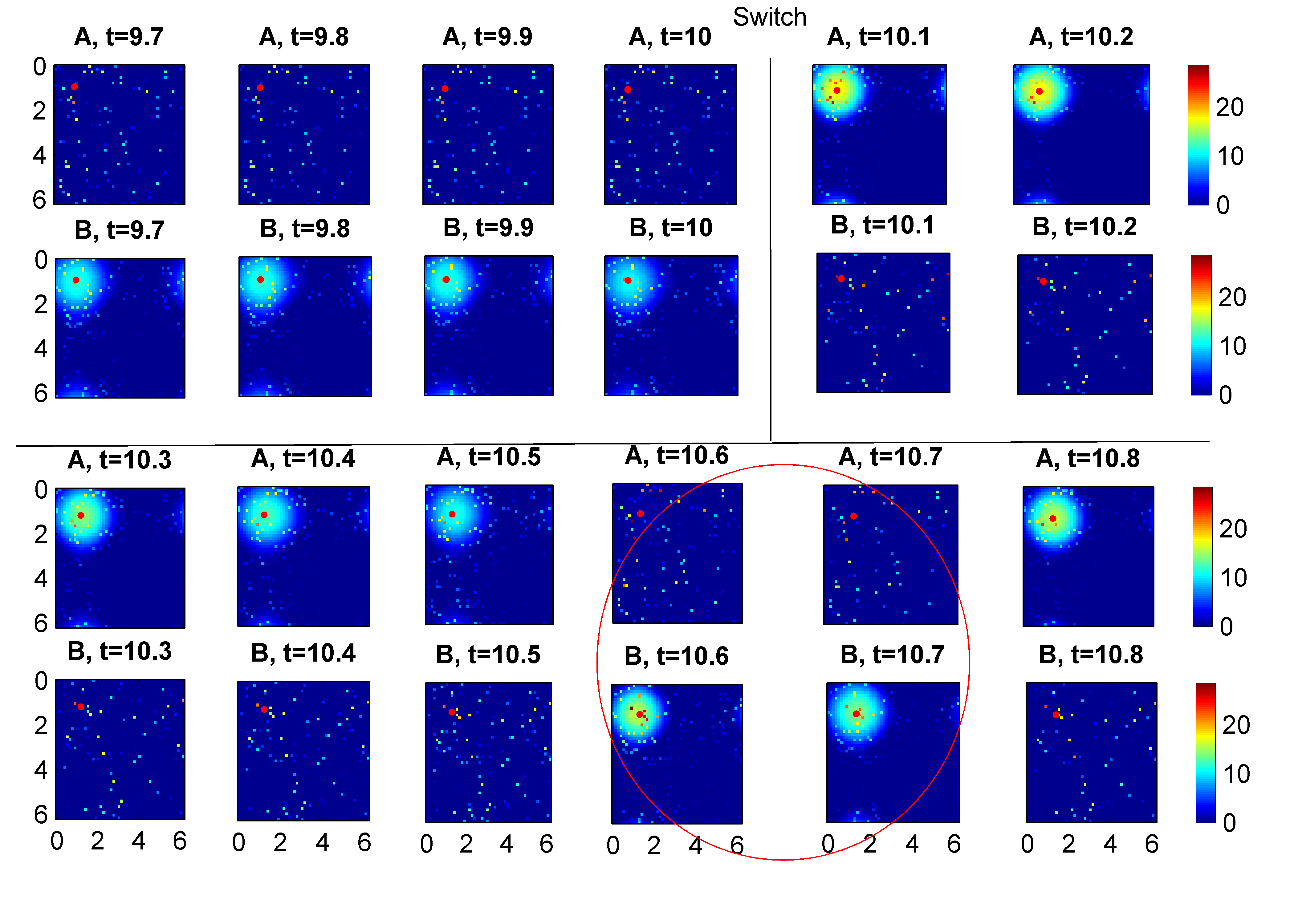

Supplement: Supplementary file 3 — Supporting Figure 1 [file HIPO-27-959-s003.tif]

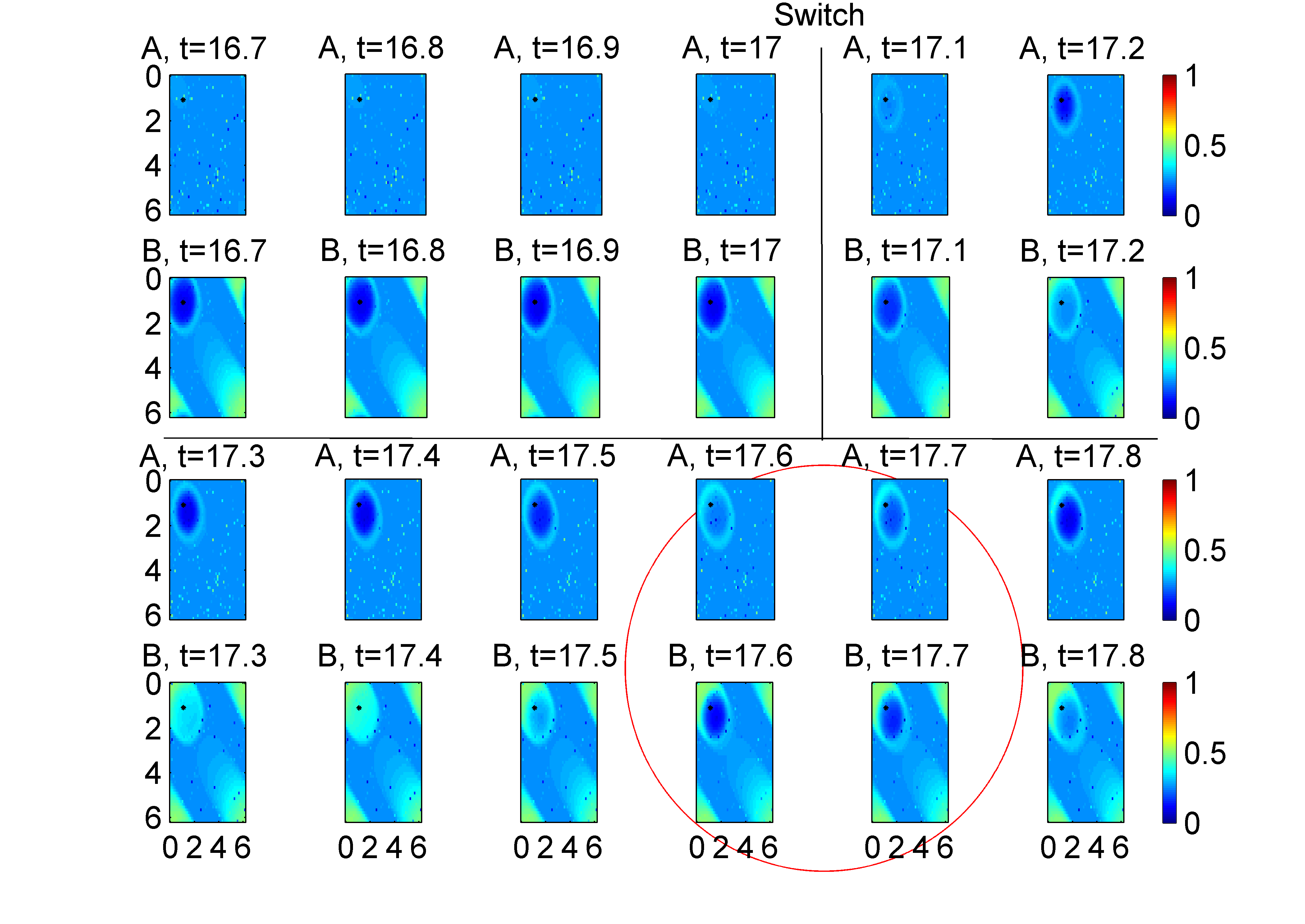

Supplement: Supplementary file 4 — Supporting Figure 2 [file HIPO-27-959-s004.tif]

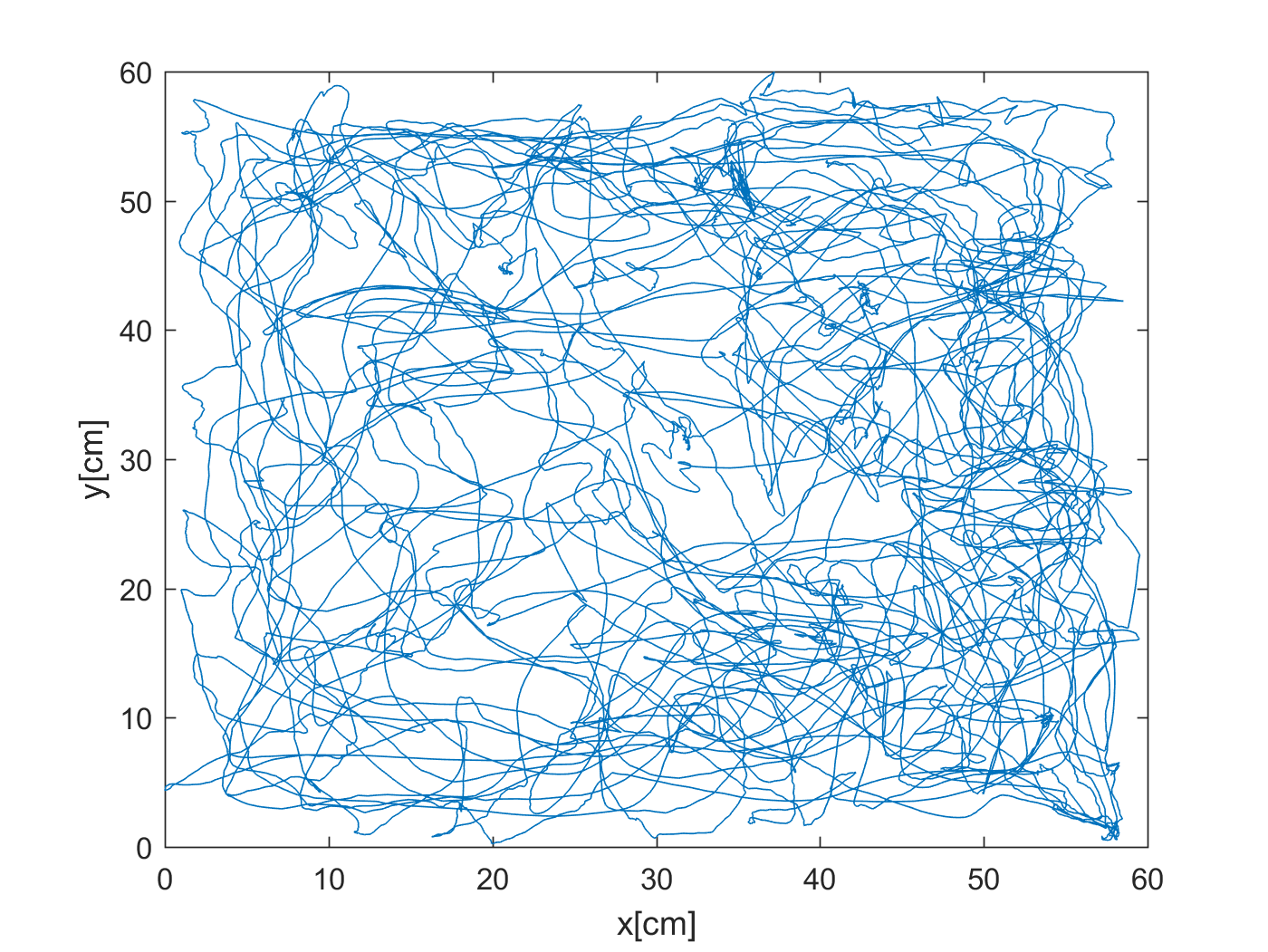

Supplement: Supplementary file 5 — Supporting Figure 3 [file HIPO-27-959-s005.tif]

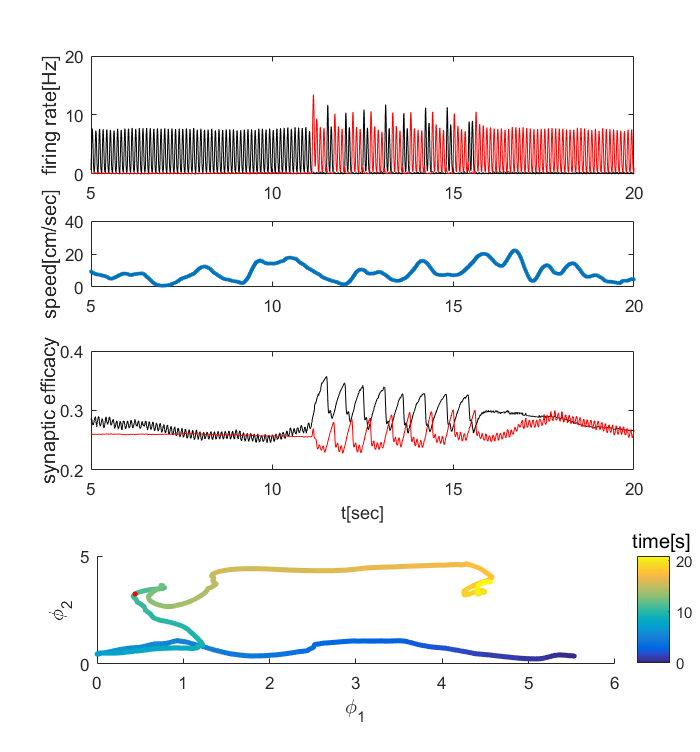

Supplement: Supplementary file 6 — Supporting Figure 4 [file HIPO-27-959-s006.tif]

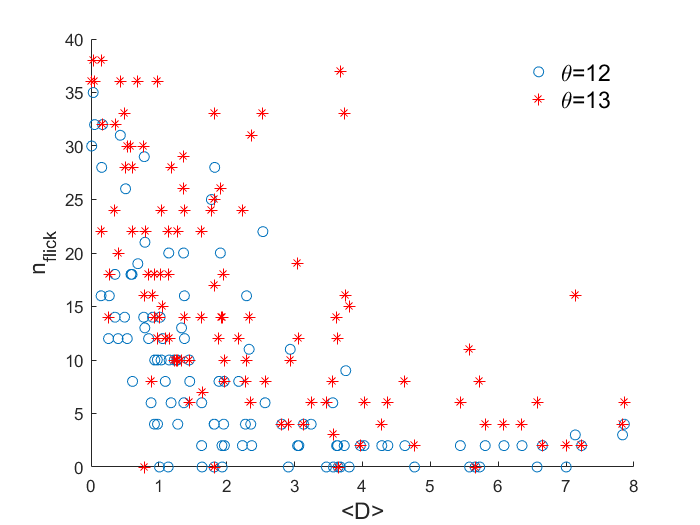

Supplement: Supplementary file 7 — Supporting Figure 5 [file HIPO-27-959-s007.tif]

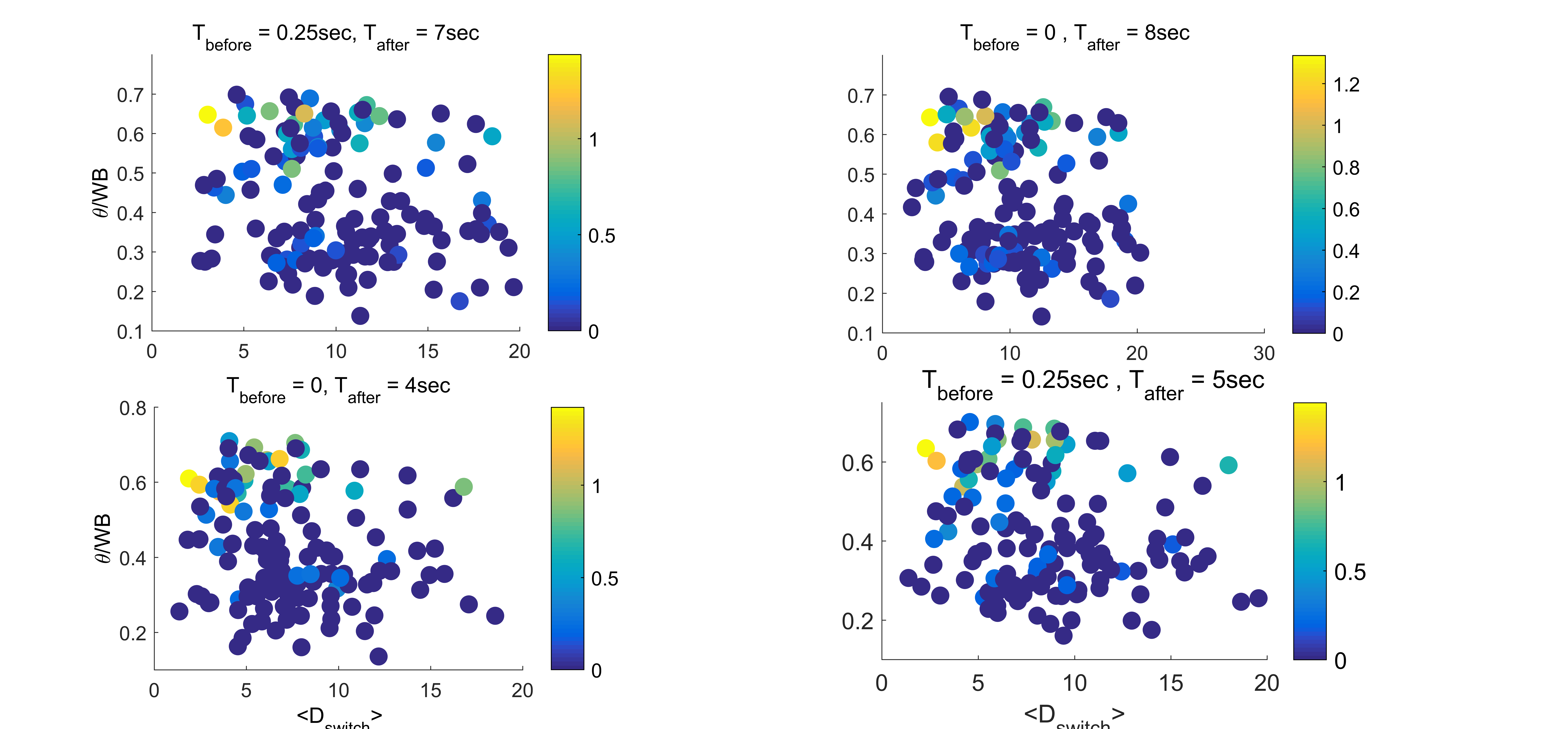

Supplement: Supplementary file 8 — Supporting Figure 6 [file HIPO-27-959-s008.tif]

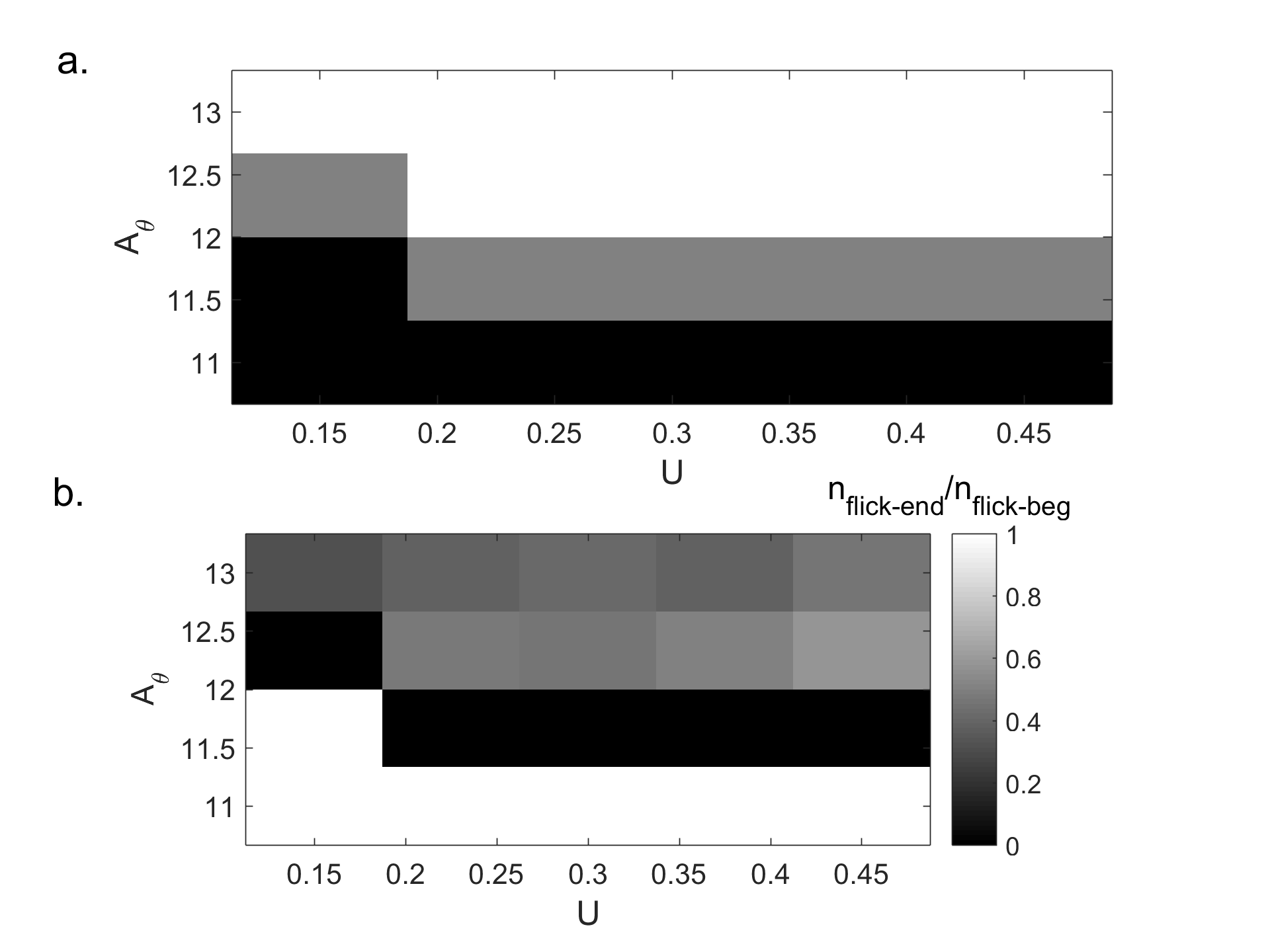

Supplement: Supplementary file 9 — Supporting Figure 7 [file HIPO-27-959-s009.tif]

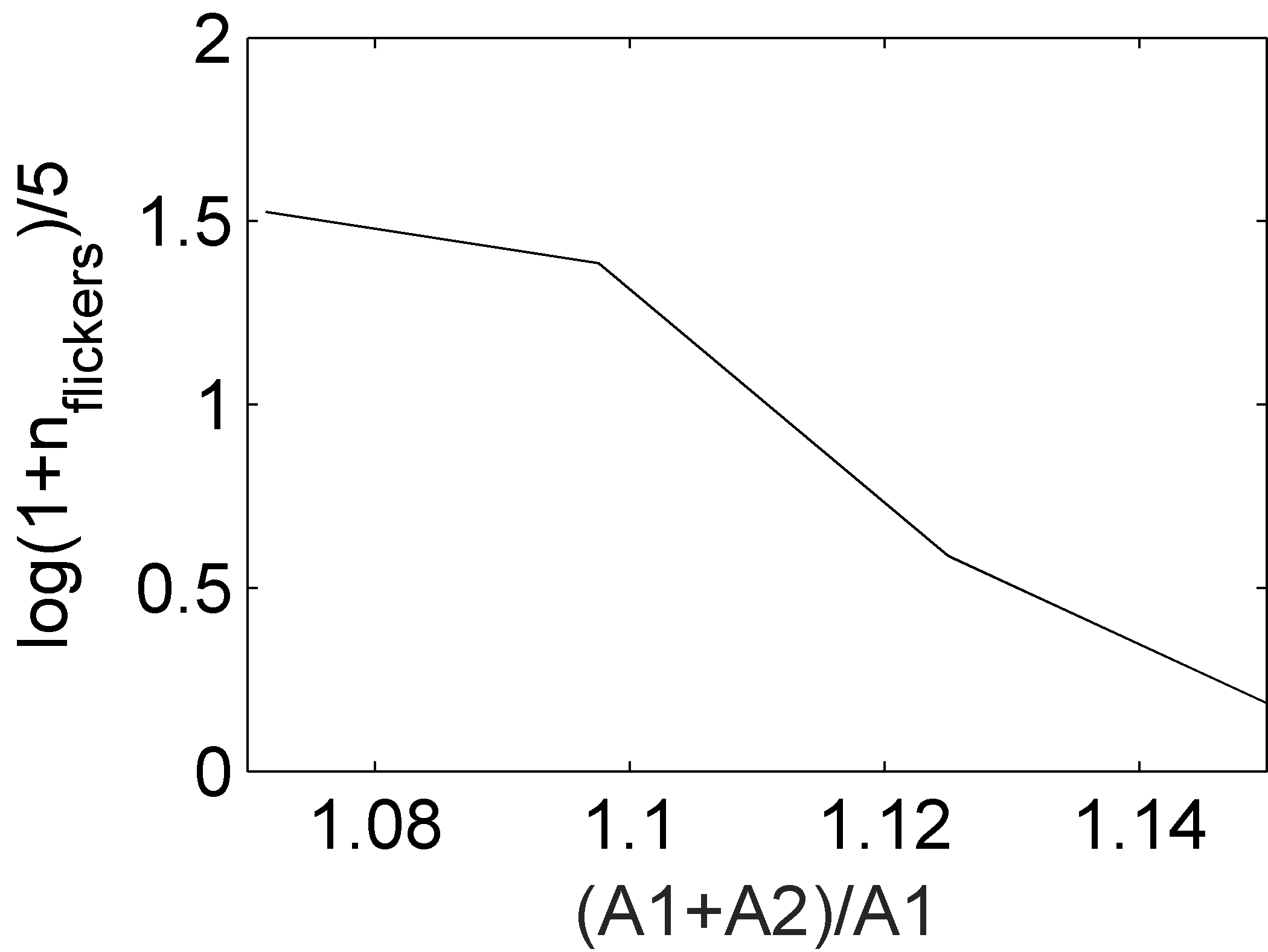

Supplement: Supplementary file 10 — Supporting Figure 8 [file HIPO-27-959-s010.tif]

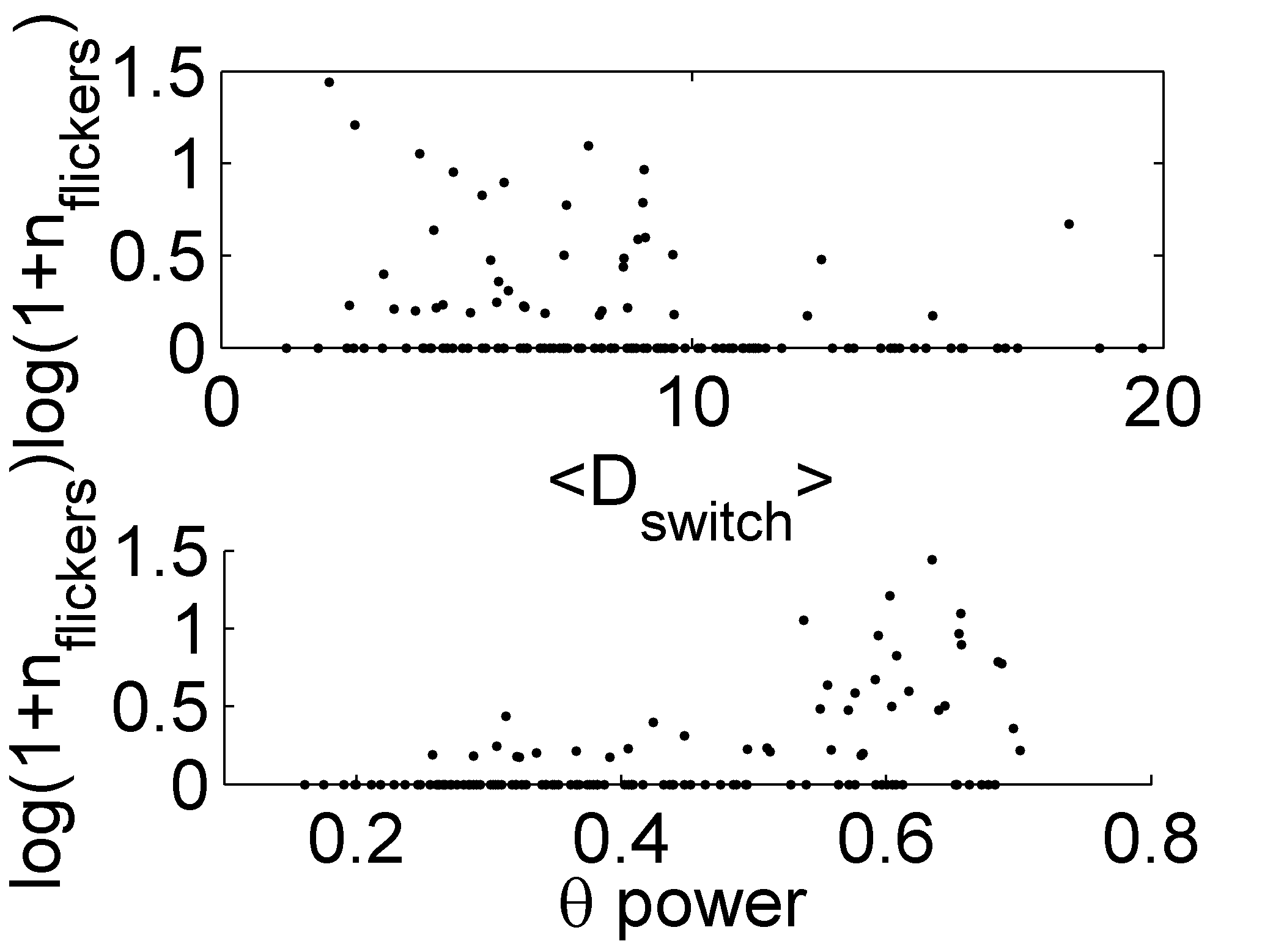

Supplement: Supplementary file 11 — Supporting Figure 9 [file HIPO-27-959-s011.tif]

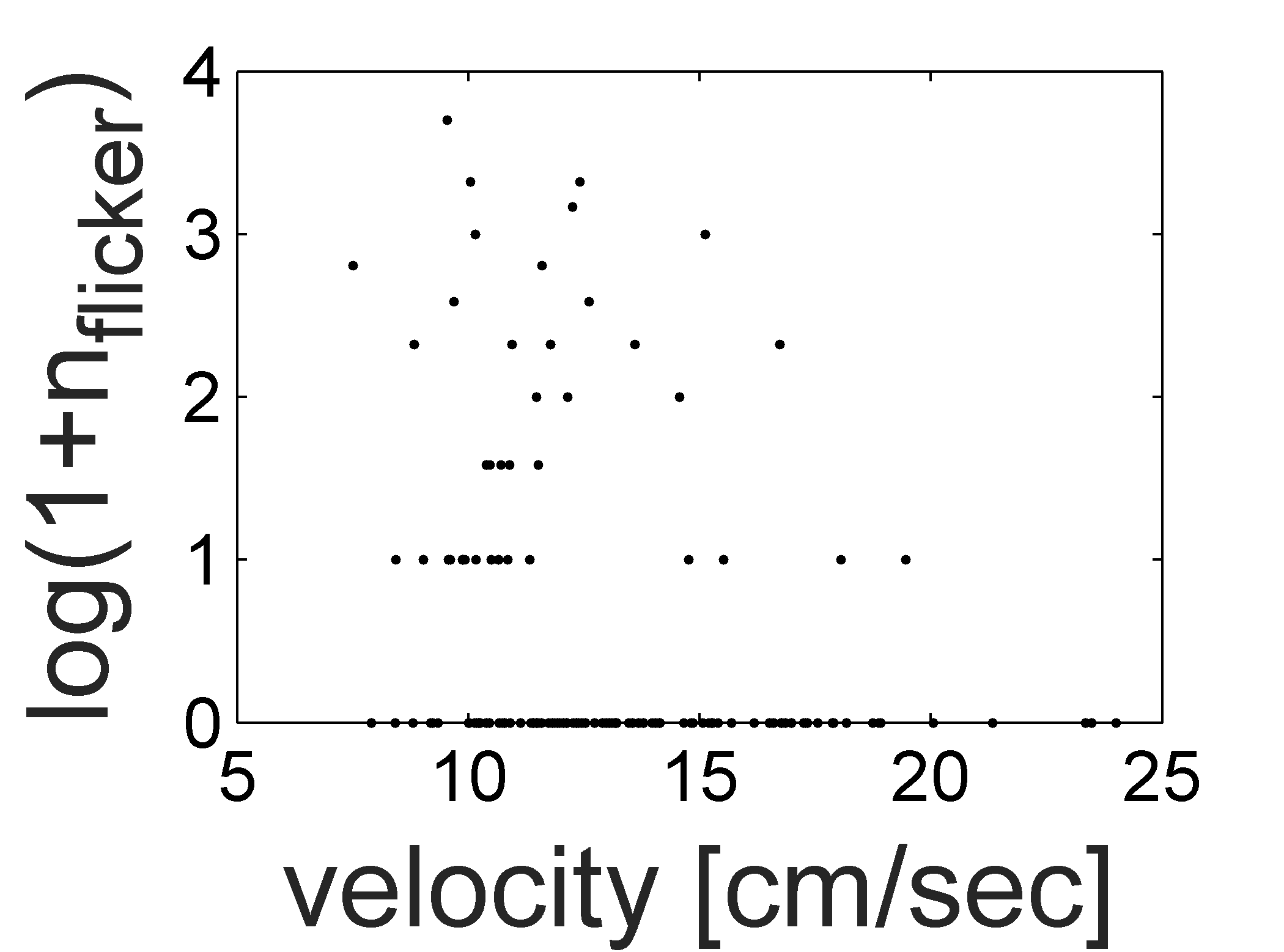

Supplement: Supplementary file 12 — Supporting Figure 10 [file HIPO-27-959-s012.tif]

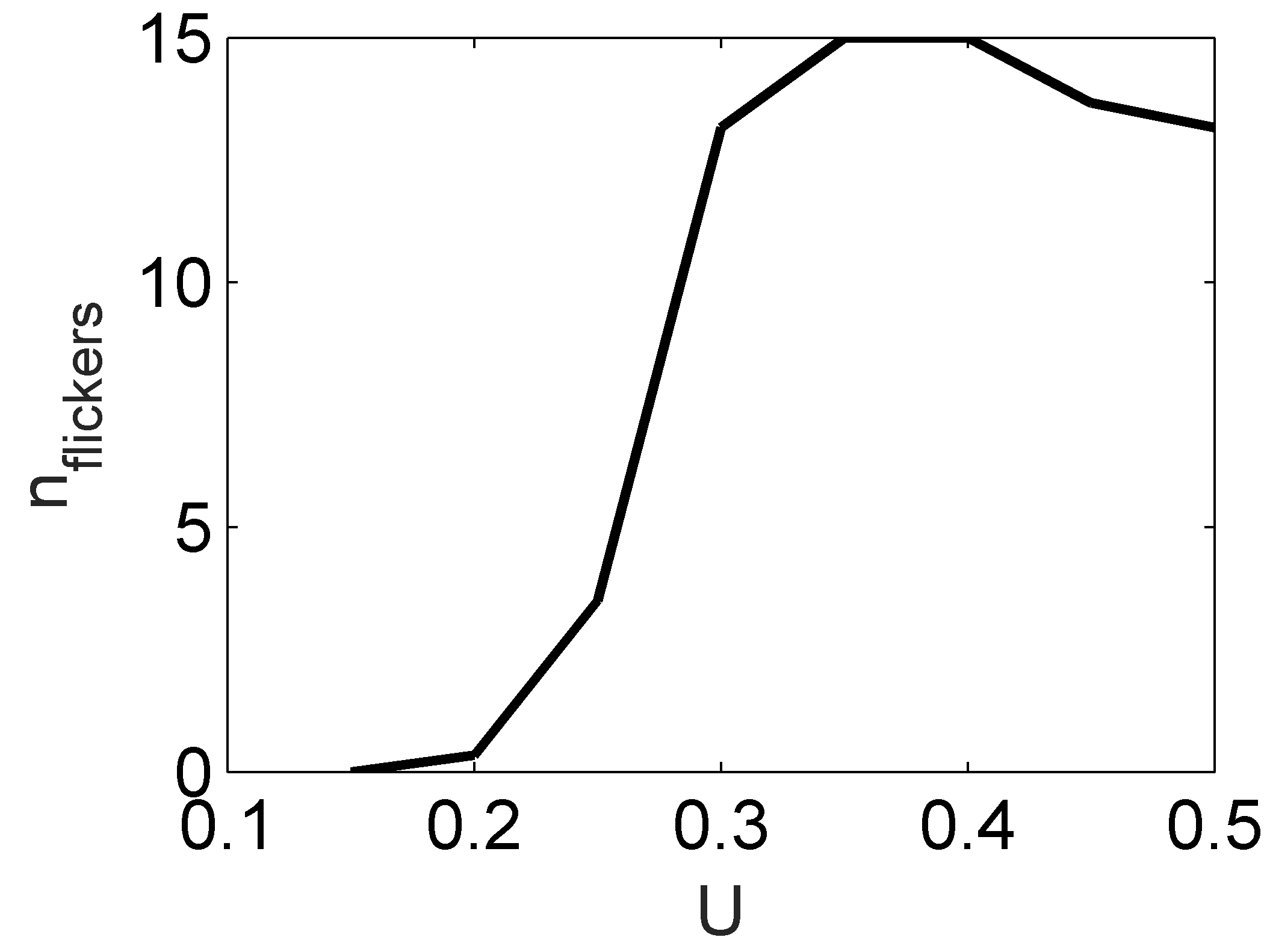

Supplement: Supplementary file 13 — Supporting Figure 11 [file HIPO-27-959-s013.tif]

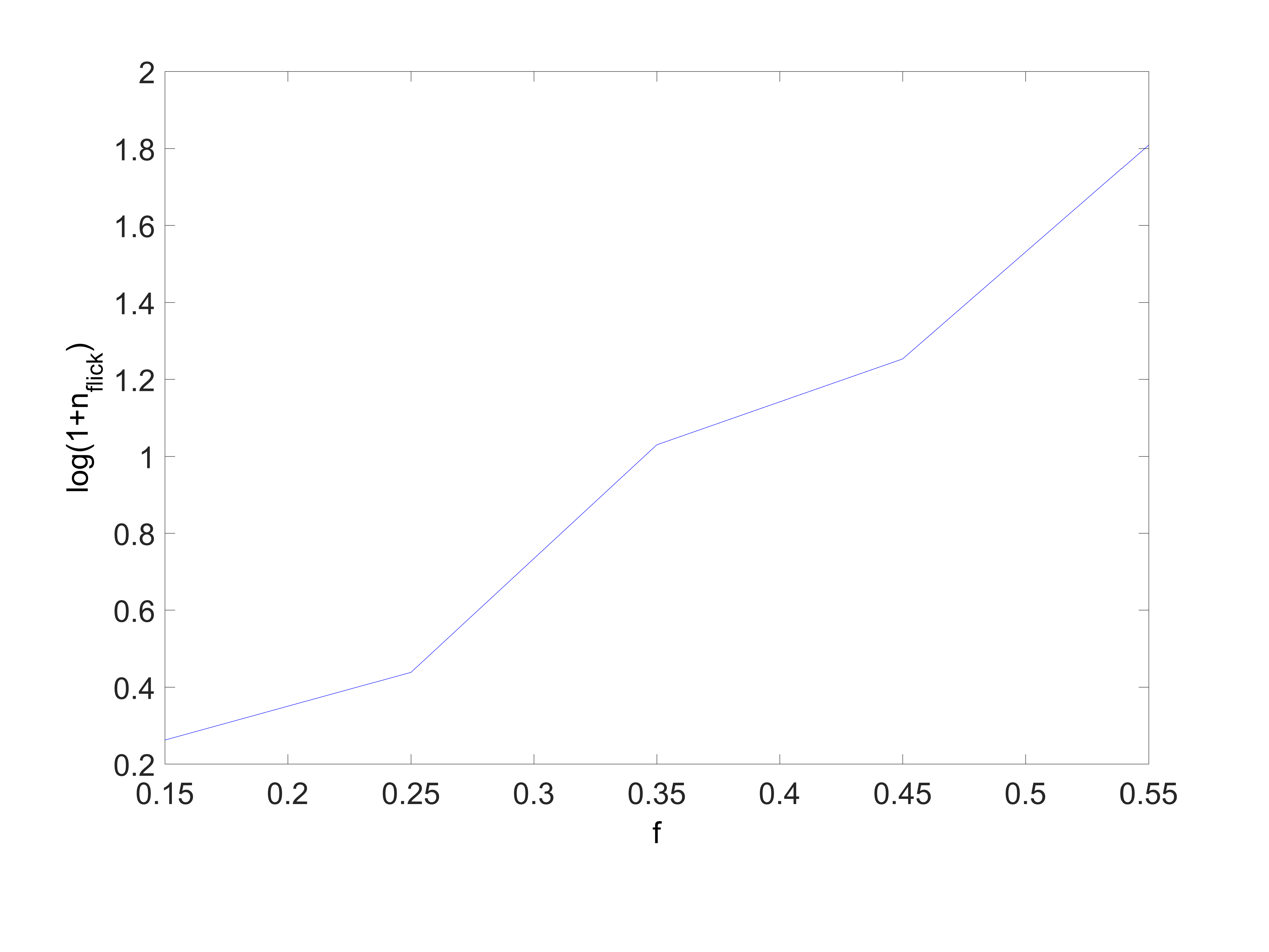

Supplement: Supplementary file 14 — Supporting Figure 12 [file HIPO-27-959-s014.tif]

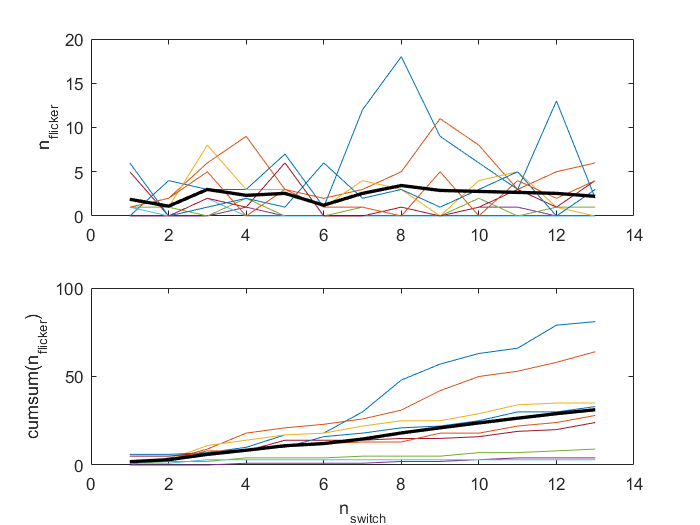

Supplement: Supplementary file 15 — Supporting Figure 13 [file HIPO-27-959-s015.tif]
